# Rheological Properties of Graphene/Polyethylene Composite Modified Asphalt Binder

**DOI:** 10.3390/ma14143986

**Published:** 2021-07-16

**Authors:** Huan-Yun Zhou, Huai-Bing Dou, Xian-Hua Chen

**Affiliations:** 1 School of Transportation, Southeast University, Nanjing 210096, China; chenxh@seu.edu.cn; 2 CCCC First Highway Reconnaissance and Design Research Institute Co., Ltd., Xi’an 710075, China; douhuaibing@126.com

**Keywords:** graphene nanoplatelets (GNPs), polyethylene (PE), asphalt binder, rheological properties, interaction mechanism

## Abstract

Aiming to improve the comprehensive road performance of asphalt binders, especially the high-temperature performance, a novel asphalt binder was prepared by compounding high-quality and low-cost polyethylene (PE) with graphene (GNPs) using a high-speed shearing machine. The rheological properties and interaction mechanism of PE/GNPs composite modified asphalt were investigated using temperature sweep (TeS), multiple stress creep recovery (MSCR), linear amplitude sweep (LAS) and Fourier transform infrared spectroscopy (FT-IR) and field emission scanning electron microscopy (FESEM). The experimental results demonstrated that GNPs and PE can synergistically improve the high-temperature performance of asphalt binders and enhance the rutting resistance of pavements; the pre-blended PE/GNPs masterbatch has good medium-temperature fatigue and low-temperature cracking resistance. Meanwhile, PE/GNPs dispersed uniformly in the asphalt matrix, and the microstructure and dispersion of premixed PE/GNPs masterbatch facilitated the asphalt modification. No new absorption peaks appeared in the FT-IR spectra of the composite modified asphalt, indicating that asphalt binders were physically modified with GNPs and PE. These findings may cast light on the feasibility of polyethylene/graphene composite for asphalt modification.

## 1. Introduction

With the growth of traffic loads and unfavorable climate, early rutting has become one of the most common diseases for asphalt pavements, and the viscoelastic properties of conventional asphalt binders can lead to permanent deformation of asphalt pavements under high-temperature and heavy loads [1]. Meanwhile, with rapid socio-economic development, a large amount of waste materials is generated worldwide every year [2]. The disposal of this waste often requires consideration of both cost-effectiveness (secondary use or recycling) and environmental issues (incineration or landfill of waste materials) [3]. In addition, a considerable amount of asphalt mixtures is produced worldwide annually for road construction; therefore, the question arises of whether it is possible to make full use of these waste materials in the road construction process. As a result, these topics have attracted the attention of researchers and engineers worldwide [4].

The concept of circular economy is gradually receiving acceptance and embrace, where it mainly refers to strategies designed (i) to reduce waste by maintaining and repairing the products being used and (ii) to utilize more reusable materials, while saving raw material consumption [5]. Hence, the utilization of waste materials for construction and infrastructure is an imperative engineering practice. Poulikakos et al. [6] reviewed the possible paths and treatments of waste materials generated by cities used for asphalt roads. They concluded that various waste materials such as glass, bitumen, concrete, wood, life-end tires, and different types of waste plastics are technically possible to be recycled in asphalt roads. Currently, waste plastics constitute a social challenge around the world. In China, more than 80% of waste plastics are burned or treated as fuel, and only a modest amount (less than 10%) is reused or recycled as feedstock [7]. These waste resources have not been fully employed by researchers, let alone used on an industrial scale. Fortunately, attempts have been made to use locally produced waste plastics for asphalt binders in the United States of America, the United Kingdom, and South Africa [8,9,10]. However, present field practices for the application of plastics in asphalt are more or less at the initial stage, and there is a significant gap in guidelines, standards, and specifications for the utilization of plastics in asphalt binders on a large scale [2].

In addition to waste reduction, the application of polyethylene (PE) may potentially minimize the binder content, which is the most expensive component of bituminous mixtures, while enhancing road performance [11]. Thermal and environmental conditions as well as traffic loading on the road pavement surface are believed to be the fundamental factors contributing to the failure of this important construction material in terms of distress. Ultimate weather conditions, such as low and high temperatures, lead to the development of pavement cracking and rutting, respectively [12]. To minimize this disease, researchers are working to improve the performance of asphalt binders.

Currently, modification of asphalt binders has been considered as a useful solution to improve the high- and low-temperature properties of asphalt mixtures [2]. Road engineers have tried to use various additive materials, such as polymers, fine fillers, etc., for asphalt binder performance enhancement, among which polyolefin modifiers, such as polyethylene (PE), polypropylene (PP), and recycled PE, have been widely used for asphalt modification due to their excellent deformation resistance [13,14]. Nevertheless, the homogeneity and storage stability of polyolefin polymer modified asphalt binders were insufficient, while they were prone to phase segregation at high temperature [15].

In recent years, nanomaterials with their unique properties, such as “small size effect”, “surface and boundary effect” and “quantum size effect”, have become the most popular research topics in many fields [16]. As a novel asphalt modifier, nanomaterials have attracted a lot of attention from pavement researchers. Research results have shown that specific nanomaterials such as nano-clay [17], nano-TiO2 [18], nano-SiO2 [19], carbon nanotubes (CNTs) [20], graphene nanoplatelets (GNPs) [21], and graphene oxide (GO) [22], can greatly improve the mechanical properties, aging resistance, fatigue resistance, and adhesion properties of asphalt binders. Although the above-mentioned nanomaterials improved some properties of asphalt binders, the improvement varied. Among them, graphene, a two-dimensional layered nanostructured material with excellent mechanical, electrical, thermal, and optical properties, has received much attention since its discovery [23].

Le et al. [24] evaluated three GNPs (M750, M850 and 4827) from different manufacturers and they found that GNPs did not significantly affect the complex modulus and phase angle of the base asphalt, but GNPs could improve the strength of asphalt binders. Li et al. [25] also observed that GNPs enhanced the mechanical strength of asphalt binders. Brcic [26] concluded that GNPs increased the rutting resistance of asphalt binders while decreasing their low-temperature thermal cracking resistance. Moreno-Navarro et al. [27] found that the incorporation of moderate amounts of graphene resulted in greater elastic response and reduced the thermal sensitivity of asphalt binders. However, the enhancement of graphene nanosheets on asphalt binders was not significant at a lower dosage, and a higher dosage of GNPs (usually higher than 1.0%) was recommended to enhance the performance of asphalt. However, the addition of a large percentage of GNPs into asphalt can significantly increase the cost of construction due to the complicated production process.

Some researchers have observed that the combination of polymers and graphene for asphalt modification greatly enhanced the road performance of asphalt binders due to the excellent small particle size of graphene, which enables it to easily mix and is more compatible with asphalt [15]. Composite graphene/polymer modification has commonly been more cost effective because they decrease the amount of polymer and graphene material while enhancing the compatibility of the polymer with the asphalt [1]. Han et al. [28] found that the addition of a certain amount of GNPs improved the storage stability of SBS modified asphalt. Meng et al. [29] concluded that GNPs could improve the aging resistance of rubber asphalt. Chen et al. [30] found that the addition of GNPs improved the high- and low-temperature properties and viscoelastic properties of rubber powder modified asphalt, while the presence of GNPs slowed down the segregation of rubber modified asphalt and improved the adhesive properties of asphalt and aggregates in the composite modified asphalt mixture. Therefore, the combination of polymers and GNPs to improve the performance of asphalt is both feasible and more attractive. Nevertheless, the conventional polymer modified asphalt has not allowed exciting results since it increases the cost of the modification [31]. On the other hand, most studies on nanomaterial/polymer composite modified asphalt usually imply a higher modifier content (4–8% by weight of the asphalt binder) to achieve the desired performance [15]. In fact, nanomaterials, especially graphene, are still very expensive. Considering the higher production cost of graphene and conventional polymers (SBS), it is essential to adapt the idea of preparation and application of graphene polymer composite modified asphalt. Unfortunately, the studies on polyolefin/GNPs composite modified asphalt binders have not been reported, and the rheological properties and interaction mechanisms of polyolefin/GNPs composite modified asphalt binders have been unclear.

**Figure 1:**
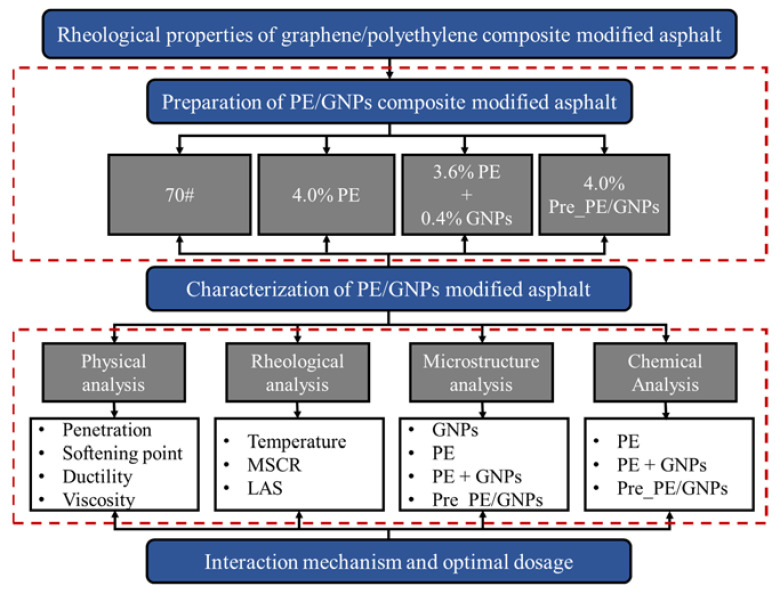
Schematic flow chart of research procedure.

To address the above-mentioned issues, the present work investigated low-cost plastic polymers (polyethylene), which can be obtained from daily waste bags [3], for asphalt modification in conjunction with low content of graphene (0.4%). The main purpose of this paper is to investigate the effect of graphene nanoplatelets on the rheological properties of polyethylene modified asphalt. The effect of adding low-cost plastic polymers with superior graphene on the performance of asphalt binder (especially low and medium temperature performance) has not been reported. On the other hand, in view of the great differences in physicochemical properties between graphene and polyethylene, two approaches were adopted to incorporate the composite modifiers, namely (1) premixed PE/GNPs masterbatch composites, and (2) adding PE and GNPs separately for the preparation of modified asphalt binders. The effect of GNPs on the physical properties of PE modified asphalt was first analyzed by the basic physical indicators (penetration, softening point, ductility). Immediately after, the influence of adding graphene nanoplatelets on the viscosity–temperature characteristics of PE modified asphalt was evaluated by using a rotational viscometer. The high- and low-temperature rheological properties of the composite modified asphalt were then investigated with the aid of a dynamic shear rheometer (DSR). Field emission scanning electron microscopy (FESEM) was also used to examine the dispersion of GNPs/PE composite modifier in the binder. Finally, Fourier transform infrared (FT-IR) spectroscopy was used to study the impact of the addition of graphene on the chemical structure of the PE modified asphalt for revealing its mechanism of action. The overall research procedure is schematically shown in Figure 1. Consequently, the current investigation may contribute to understand the performance characteristics of new composite modified asphalt in order to improve the design approach of asphalt mixtures with high performance.

## 2. Materials and Methods

### 2.1. Materials

**Figure 2:**
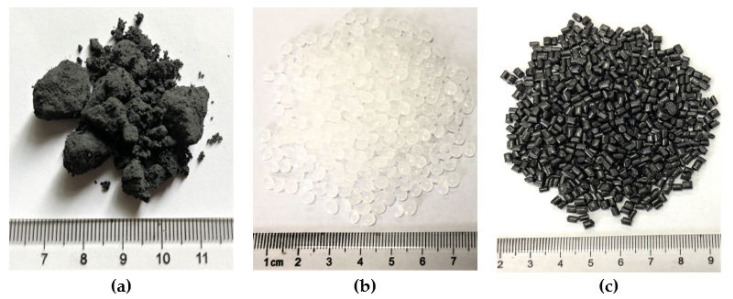
The images of raw materials used in the study. (**a**) GNPs powders, (**b**) PE particles, (**c**) Pre_GNPs/PE particles.

The raw materials used in this experimental study included base asphalt, multilayer graphene nanoplatelets (GNPs), low-density polyethylene (PE), and pre-blended PE/GNPs masterbatch composites (Haichuan New Material Co., Ltd., Shenzhen, China). A Pen-70 base asphalt binder was employed; its physical properties are listed in Table 1. The properties of GNPs are presented in Table 2. Polyethylene is a commercial linear low-density PE particle; its properties are summarized in Table 3. Pre-mixed PE/GNPs masterbatch composite was a composite material in which GNPs dispersed in a polyethylene with a 10% GNPs content (by weight of PE). The GNPs and PE used in a separate addition were identical to the material in the pre-mixed PE/GNPs masterbatch. The above raw materials are depicted in Figure 2.

### 2.2. Preparation of PE/GNPs Modified Asphalt Binder

Good dispersion of the nano-sized additives in the asphalt matrix is a crucial and challenging step in the preparation of nano-modified asphalt binders. Referring to the report of relevant researchers [16], a high-speed shear emulsifier was employed to prepare PE/GNPs composite modified asphalt binders. Different addition procedures were adopted for each of the two different composite additives.

(1) Pre-blended PE/GNPs masterbatch composite (pure PE particles) modified asphalt binder: first, the pre-blended PE/GNPs masterbatch composite (pure PE particles) (4.0%, by weight of the base asphalt) was manually mixed into the molten base asphalt while slowly stirring with a glass rod until the solid modifier was completely dissolved. Then, the mixture containing pre-blended PE/GNPs masterbatch (pure PE particles) and base asphalt was warmed up to 170 °C and sheared with a high-speed shear mixer at a constant speed of 5000 r/min for 30 min. The mixture was then stirred at 170 °C with a mechanical stirrer at 1000 r/min for 60 min. The resulting 4.0% Pre_PE/GNPs or 4.0% PE modified asphalt binders were synthesized.
(2) Combined addition of PE granules and GNPs powder modified asphalt binder: in order to ensure that the amount of modifier in PE+GNPs composite modified asphalt is exactly the same as that in PE/GNPs masterbatch (pure PE granules) (4.0% of the mass of the matrix asphalt), and at the same time based on the mass percentage of GNPs in the premixed PE/GNPs masterbatch is 10% (mass ratio of the masterbatch), thus determining that the content of PE in the PE+GNPs modified asphalt is 4.0% × 90% = 3.6%; the content of GNPs is 4.0% × 10% = 0.4%; that is, the amount of modifier in the PE+GNPs composite modified asphalt is 3.6% PE + 0.4% GNPs.

Since GNPs material is a light and fluffy material, it tends to fly and drift during the mixing process, resulting in mass loss. Therefore, firstly, an appropriate amount of GNPs (0.4%, by the weight of the base asphalt) was mixed into the molten base asphalt in batches using a glass rod until GNPs was completely dissolved in the matrix asphalt. Then, the GNPs–asphalt mixture was mixed with PE (3.6%, by weight of the base asphalt) for 30 min at 5000 r/min using a high-shear emulsifier, and finally, the GNPs-PE-asphalt mixture was mixed with a stirrer for 60 min at 1000 r/min. The above steps were performed at a constant temperature of 170 °C.

For comparison, the unmodified asphalt binder samples were subjected to the same preparation procedures as described above. The rheological properties of each asphalt samples were measured three times in parallel and the average value was used as the test result.

### 2.3. Characterization and Measurement

#### 2.3.1. Physical Properties Testing

The Brookfield viscometer (DV-II+Pro, BROOKFIELD ENGINEERING LABORATORIES, INC., MA, USA) was used to quantitatively evaluate the viscosity properties of PE/GNPs composite modified asphalt binder samples under different temperatures. In addition, the needle penetration, softening point, and ductility of the modified asphalt binder were also measured according to the standard test procedure of “Test Procedure for Bitumen and Bituminous Mixtures for Highway Engineering” (JTG E20-2011) [32]. Each combination of asphalt samples was tested three times in parallel under the same conditions to ensure the reproducibility of the results.

#### 2.3.2. Temperature Scanning (TeS) Test

An air-bearing strain-controlled dynamic shear rheometer (HR-1, TA instruments, New Castle, DE, USA) was employed to characterize the viscoelastic properties of the PE/GNPs composite modified asphalt binder. The temperature sweep test was performed with a continuous heating rate of 1 °C/min, a fixed frequency of 10 rad/s, a temperature range of 40–82 °C, and an interval of 6 °C; the temperature sweep procedure was adopted in strain-controlled mode (1.25%). All combination asphalt samples were tested three times in parallel under the same condition to ensure the reproducibility of the results.

#### 2.3.3. Multiple Stress Creep Recovery (MSCR) Test

To more accurately characterize the high-temperature stability of the composite modified asphalt binder, the multiple stress creep recovery (MSCR) test recommended by AASHTO [33] was conducted at 100 Pa and 3200 Pa. The MSCR test was performed for 10 cycles at each stress level, which consisted of 1 s loading and 9 s unloading for each loading cycle. Each loading cycle consisted of 1 s loading and 9 s unloading, and 60 °C was selected as the test temperature to better simulate the road field conditions. All combination asphalt samples were tested three times in parallel under the same condition to ensure the reproducibility of the results.

#### 2.3.4. Linear Amplitude Scanning (LAS) Test

In order to evaluate the fatigue resistance of asphalt binder, the linear amplitude sweep (LAS) test was performed by the strain-controlled loading method based on DSR according to the test protocol of AASHTO [34]. The stresses and strains obtained from the LAS test data were nonlinearly matched using the continuous damage model VECD to obtain the damage characteristic curves of the asphalt binder and then determine the fatigue life of PE/GNPs composite modified asphalt. Based on the analysis of the actual service temperature of asphalt pavement, 25 °C was selected as the test temperature. All the combination asphalt samples were tested three times in parallel under the same pieces to ensure the reproducibility of the results.

#### 2.3.5. Field Emission Scanning Electron Microscope (FESEM) Characterization

The dispersion and microstructure of graphene and PE in asphalt matrix was investigated by using a field emission scanning electron microscope (FEI Quanta 250, FEI company, Hillsboro, OR, USA). FESEM sample preparation consists of asphalt film preparation and spraying gold powder procedures. The samples were first heated at 170 °C for 30 min and meanwhile ensured to be melted and could be poured into FESEM molds containing about 0.5 g of bitumen binder. The filled ESEM molds were then placed on a hot plate (110 °C) to flatten the sample surface for two minutes [35]. Finally, an appropriate amount of gold powder was uniformly sprayed onto the above smoothed sample surface. Immediately afterwards, microscopic images of the various asphalt binders were obtained by exposing the composite modified asphalt binders to the electron beam for disposal at 10 keV electron voltage and 3.0 dot size for a few minutes [2].

#### 2.3.6. Fourier Transform Infrared Spectroscopy (FT-IR) Testing

Fourier transform infrared (FTIR) spectroscopy is a powerful technique for identifying the chemical structure of materials. In order to characterize the changes in chemical properties of asphalt binders before and after modification, the chemical structure and functional groups of PE/GNPs composite modified asphalt samples were determined with the help of FT-IR. In the test, asphalt samples coated with potassium bromide (KBr) were placed in an FT-IR instrument (Nicolet 6700, Thermo Fisher Scientific Inc., Waltham, MA, USA) [2]. The prepared specimens were scanned with an FTIR spectrometer and the IR spectral data of the asphalt binder were recorded in the wave number range of 4000–600 cm^−1^.

## 3. Results and Discussions

### 3.1. Physical Properties

#### 3.1.1. Three Major Indicators Data Analysis

**Figure 3:**
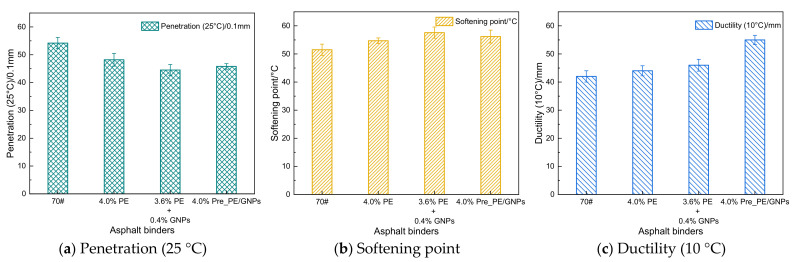
The physical properties of asphalt binder samples. (**a**) Penetration (25 °C), (**b**) Softening point, (**c**) Ductility (10 °C).

It is generally believed that the softening point reflects the high-temperature softening performance of asphalt binder, the ductility represents the ductility characteristics of asphalt binder at low-temperature conditions, and the penetration indicates the relative hardness and consistency of asphalt [15]. The physical properties of PE/GNPs composite modified asphalt binder and control asphalt samples are shown in Figure 3. Compared with the processed base asphalt (70#), the PE/GNPs composite modified asphalt binder (3.6% PE + 0.4% GNPs and 4.0% Pre_PE/GNPs, same below) exhibited a higher softening point (high-temperature property) and ductility (low-temperature property) (Figure 3a,b) and lower penetration (hardness of asphalt) (Figure 3c). The above results indicated that PE/GNPs composites improved the high-temperature properties of asphalt samples. Meanwhile, the PE/GNPs composite modifier also enhanced the ductility and flexibility of the base asphalt binder. In other words, the incorporation of PE/GNPs composite modifier benefited both the high-temperature performance and low-temperature flexibility of the asphalt binder. Compared with PE modified asphalt, the softening point and ductility of PE/GNPs composite modified asphalt increased, while the penetration decreased. The experimental data demonstrated that the addition of a small amount of GNPs to PE modified asphalt would harden the asphalt binder, but the hardening effect of GNPs did not deteriorate the low-temperature flexibility of PE modified asphalt. The above analysis confirmed that PE and GNPs have a synergistic effect in improving the high-temperature performance of asphalt binder, while the PE/GNPs composite modifier contributed to enhancing the low-temperature cracking resistance of asphalt.

#### 3.1.2. Viscosity–Temperature Characteristics

**Figure 4:**
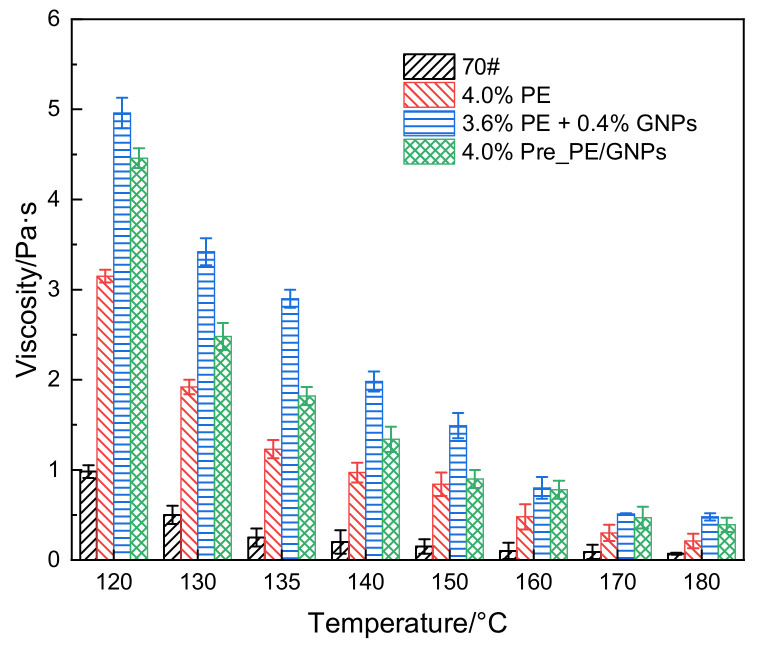
Viscosity–temperature trend of PE/GNPs composite modified asphalt and control asphalt samples.

As a typical viscoelastic construction material, the viscosity characteristics of asphalt are closely related to the application temperature; a lower viscosity is beneficial for field construction [22]. Figure 4 shows the rotational viscosity of PE/GNPs composite modified asphalt samples and control asphalt at different temperatures. The introduction of the appropriate amount of PE/GNPs composite modifier led to an increased rotational viscosity of the base asphalt. The viscosity of the PE/GNPs composite modified asphalt binder was generally higher than that of the PE modified asphalt (with the same PE content in the base asphalt) in the testing temperature range because GNPs acted as fillers and increased the friction between the asphalt molecules in the binder [36]. However, the viscosity of the premixed 4.0% Pre_PE/GNPs modified asphalt binder was lower than that of the asphalt binder with separate additions of CNTs and PE (3.6% PE + 0.4% GNPs), which may be attributed to the adsorption of GNPs in the asphalt [15], thus increasing the viscosity of the 3.6% PE + 0.4% GNPs modified asphalt binder.

It is expected that the viscosity of the asphalt binder gradually decreases as the temperature increases. From the perspective of construction, the viscosity of the asphalt binder should be less than 3.0 Pa·s at 135 °C [37]. The viscosity test results showed that the workability of all PE/GNPs composite modified bituminous binders could easily meet the requirement. It should be noted that the addition of PE/GNPs increased the viscosity of the binder implying higher mixing and compaction temperatures (not less than 170 °C, corresponding to a viscosity of less than 0.3 Pa·s [21]) as well as energy consumption.

### 3.2. Rheological Properties

#### 3.2.1. Temperature Scan Results

In general, asphalt binder has a large mechanical strength (complex modulus) at low-temperature conditions showing typical elastic properties; as the temperature increases, the asphalt binder gradually softens, the complex modulus decreases and tends to have a viscous flow; when the temperature is further increased, the internal molecular thermal movement of the asphalt binder is enhanced, and the overall performance exhibits typical plastic flow, at which time the risk of high-temperature rutting damage is higher [28]. The phase angle reflects the strain hysteresis in the asphalt binder under external stress, i.e., it represents the ratio between the visco-elastic components in the asphalt; the larger the phase angle represents the more viscous components in the asphalt, the greater the possibility of non-recoverable deformation [25].

**Figure 5:**
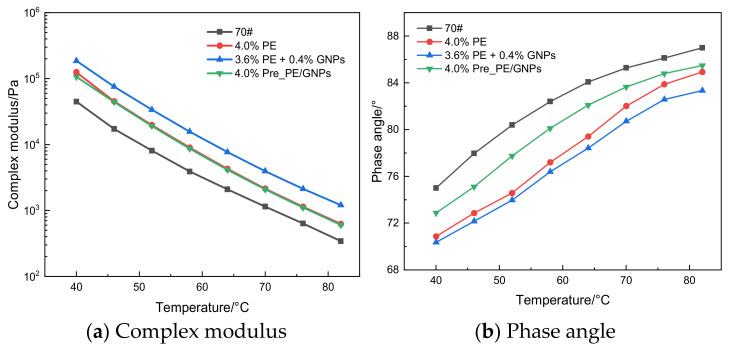
Variation of viscoelastic mechanical response of asphalt binders with temperature. (**a**) Complex modulus, (**b**) Phase angle.

Figure 5 shows the temperature sweep test results of PE/GNPs composite modified asphalt binder and the control asphalt binder. As shown in Figure 5a, the complex modulus of the asphalt binder containing PE/GNPs additive increased significantly in the temperature range of 40–82 °C compared with the base asphalt binder, especially in the relatively high temperature range. A simultaneous comparison of the complex shear modulus of the two PE/GNPs composite modified asphalt binders revealed that the use of PE and GNPs in combination (3.6% PE + 0.4% GNPs) imparted greater stiffness and smaller phase angle to the asphalt binder (Figure 5b), which indicated that the combined use of PE and GNPs could enhance the elastic component of the asphalt, thus making the asphalt binder resistance to larger deformation, and this “hardening and strengthening” effect was attributed to the excellent mechanical strength of GNPs [27]. At the same time, the complex modulus values of pre-blended PE/GNPs masterbatch modified asphalt (4.0% Pre_PE/GNPs) were almost comparable compared to PE modified asphalt, but the phase angle was relatively larger, probably because the pre-blending process weakened the rigidity of PE and increased the plastic flow of PE particles in the asphalt matrix.

#### 3.2.2. Multi-Stress Creep Recovery (MSCR) Test Results

**Figure 6:**
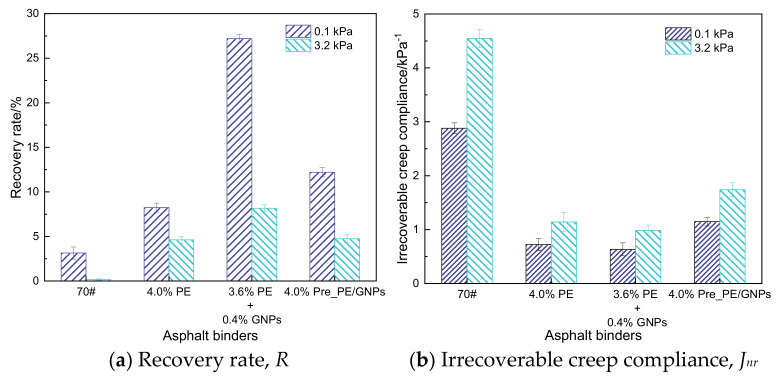
Test parameters of multiple stress creep recovery of asphalt binders. (**a**) Recovery rate, *R*, (**b**) Irrecoverable creep compliance, *J_nr_*.

Researchers generally agree that the multiple stress (0.1 kPa and 3.2 kPa) creep recovery (MSCR) test can better characterize the high-temperature stability of asphalt binders, and its main evaluation indicators include the deformation recovery rate *R* and the irrecoverable creep compliance *Jnr* of asphalt binder samples [28]. The *Jnr* value is closely related to the high-temperature rutting performance of asphalt pavement, and it is considered that the smaller the irrecoverable creep compliance, the larger the deformation recovery rate R corresponds to the larger the delayed elastic deformation of asphalt binder, the stronger its resistance to high-temperature irrecoverable deformation, and the smaller the occurrence of rutting distress [1]. As shown in Figure 6, the 3.6% PE + 0.4% GNPs modified asphalt binder showed the largest deformation recovery rate *R* at both stress levels (Figure 6a) and the smallest irrecoverable creep compliance *Jnr* (Figure 6b), while for the 4.0% PE modified asphalt and 4.0% Pre_PE/GNPs modified asphalt exhibited comparable deformation recovery rates *R* (4.0% Pre_PE/ GNPs was slightly greater than that of 4.0% PE), the latter yielded a relatively larger amount of irrecoverable creep compliance *Jnr*. This implied that the combination of PE and GNPs could significantly improve the elastic recovery performance of the base asphalt and enhance the high-temperature rutting resistance of the asphalt binder.

Table 3 shows that the stress sensitivity (*J_nr-diff_*) of PE/GNPs composite modified asphalt prepared by different mixing processes differs somewhat: the smaller the *J_nr-diff_* is theoretically, the less sensitive the asphalt binder is to loading stress, the more it tends to be elastomeric, and the better the high-temperature stability [33]. The PE/GNPs masterbatch modified asphalt prepared by the premixing process exhibited the least stress sensitivity, suggesting that it was able to withstand greater stress levels and may be more suitable for paving in complex traffic sections. This improved high-temperature stability was attributed to the mechanical strength of PE and GNPs themselves, while the premixing process promoted better synergy between the two within the asphalt matrix. Although the premixed PE/GNPs masterbatch reduced the stiffness of the PE modified asphalt binder to some extent, its excellent deformation recovery ability was not deteriorated, which may also imply that the PE/GNPs masterbatch modified asphalt possessed a better low-temperature stress relaxation capability.

#### 3.2.3. Linear Amplitude Scanning (LAS) Test Results

**Figure 7:**
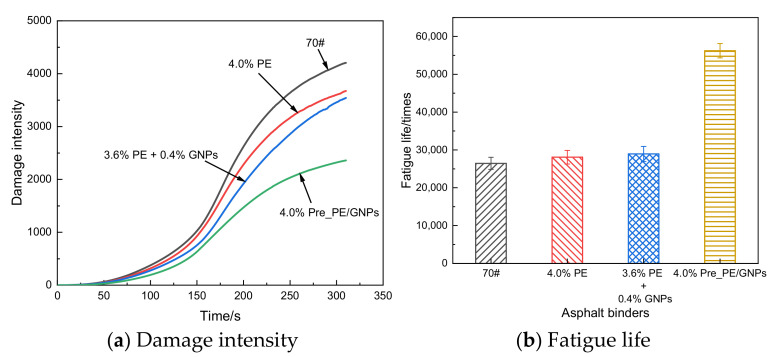
Fatigue test parameters of asphalt binder samples. (**a**) Damage intensity, (**b**) Fatigue life.

To further evaluate the fatigue resistance of PE/GNPs composite modified asphalt at medium temperature (25 °C), a linear amplitude sweep (LAS) test was conducted according to the AASHTO test protocol. The test data were processed based on the viscoelastic continuous damage (VECD) model theory [34], and the test results were presented in Figure 7. The fatigue damage of the base asphalt (70#) was the largest (Figure 7a), indicating that its fatigue damage increased sharply with the increase of loading amplitude; the damage growth rate of the asphalt binder gradually slowed down after the addition of PE/GNP composite modifier, suggesting that the introduction of the appropriate amount of PE/GNPs can inhibit or slow down the propagation of fatigue cracks within the asphalt binder, thus improving its fatigue resistance. In comparison with PE modified asphalt, the presence of GNPs can effectively reduce the damage of PE modified asphalt and thus enhance its fatigue life. It should be noted that the modified asphalt prepared by adding PE and GNPs independently (3.6% PE + 0.4% GNPs) could decrease the fatigue damage growth rate of PE modified asphalt binder, but the final damage of the two was close to each other. Although the fatigue damage growth rate of PE modified asphalt binder was slowed down, the final damage of both was close to the same extent, i.e., the fatigue life of both was approximately equal (Figure 7b), which indicated that the addition process separately only partially exploited the effectiveness of GNPs and wasted the excellent mechanical strength potential of GNPs. However, the pre-blended PE/GNPs masterbatch composite asphalt exhibited great advantages in terms of both fatigue damage and fatigue life (minimum damage and maximum fatigue life), which may be attributed to the fact that the pre-blending process balanced both the “stiffness reduction and toughening” of PE and the uniform distribution of GNPs, thus greatly enhancing the fatigue resistance of the asphalt binder.

### 3.3. Microscopic Morphological Characterization

**Figure 8:**
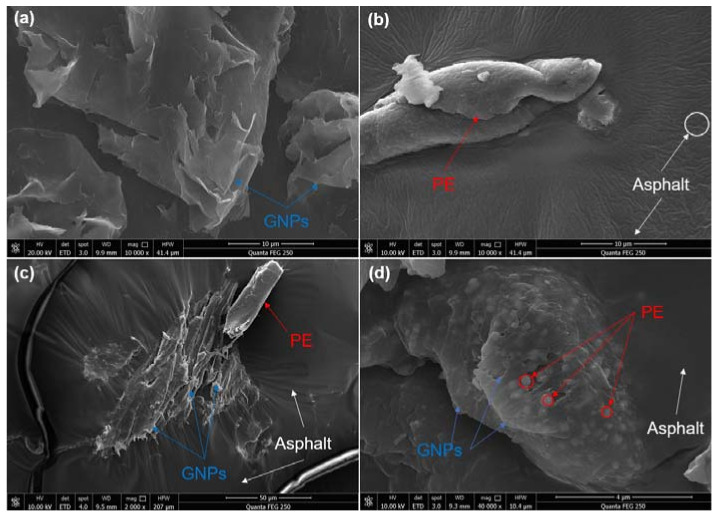
The SEM images of GNPs and various modified asphalt binders: (**a**) GNPs powder, (**b**) PE modified asphalt, (**c**) PE+GNPs modified asphalt, and (**d**) premixed PE/GNPs modified asphalt.

In order to further characterize the microstructure of PE/GNPs modified asphalt, the micro-texture of several modified asphalt binders was observed by using FESEM. Figure 8 presents the SEM topography of GNPs and PE, PE+GNPs and premixed PE/GNPs modified asphalt. As shown in Figure 8a, the light and translucent sheets represented GNPs, while GNPs inevitably exhibited some stacking based on strong van der Waals forces [15]. One can see from Figure 8b, the addition of PE made the surface of the asphalt matrix rough and wrinkled, which might make the base asphalt brittle and hard due to the intrinsic hardness of PE polymer.

As can be observed from Figure 8c, when GNPs and PE were added separately into the base asphalt binder, GNPs nanoparticles gradually adsorbed to the PP molecules and thus formed a quasi-composite system in the binder, which further enhanced the stiffness of the asphalt matrix. However, GNPs connected with PE only by a simple physical lap in the binder, leading to an unsystematic increase in the flexibility of the base asphalt. Accordingly, this might emerge local stress concentration and even produced a GNPs aggregation, which may weaken the low-temperature anti-cracking of PE+GNPs modified asphalt. Meanwhile, the swelling of PE in asphalt caused a reduction in the lightweight component of the asphalt binder, resulting in a crumpled substrate surface, which in turn may affect the aging resistance of the asphalt binder. Therefore, the microstructure of PE+GNPs modified asphalt explained the increased complex modulus and rutting resistance obtained from DSR tests and the MSCR test, respectively.

Nevertheless, the PE and GNPs can be fully integrated through the premixing process, and form a true composite system, as shown in Figure 8d. the premixed PE/ GNPs composite modifier with a uniform and small volume distribution was more conducive to the connection with the asphalt molecules [2]. Simultaneously, the rigid PE particles were wrapped around the GNPs in the composite system, making the premixed PE/GNPs structure more compact and flexible (since the nano-properties of GNPs conferred more flexibility and toughness to the PE molecular chains). Therefore, GNPs and PE synergistically enhanced the modulus, recovery rate and low-temperature flexibility of the base asphalt.

Obviously, the biggest advantage of the premixed PE/GNPs composite was that once the PE modifier was evenly dispersed into the asphalt, the GNPs was homogeneously dispersed as well without worrying about the agglomeration and drift caused by its huge specific surface area and the nano-effect. As a result, a secondary enhancement of the mechanical properties for base asphalt was achieved. Interestingly, the surface of the PE/GNPs composite modified bitumen appeared to be smoother and flatter, which might imply the anti-aging potential and application for composite modified asphalt to some degree.

### 3.4. Fourier Transform Infrared Spectroscopy Analysis

**Figure 9:**
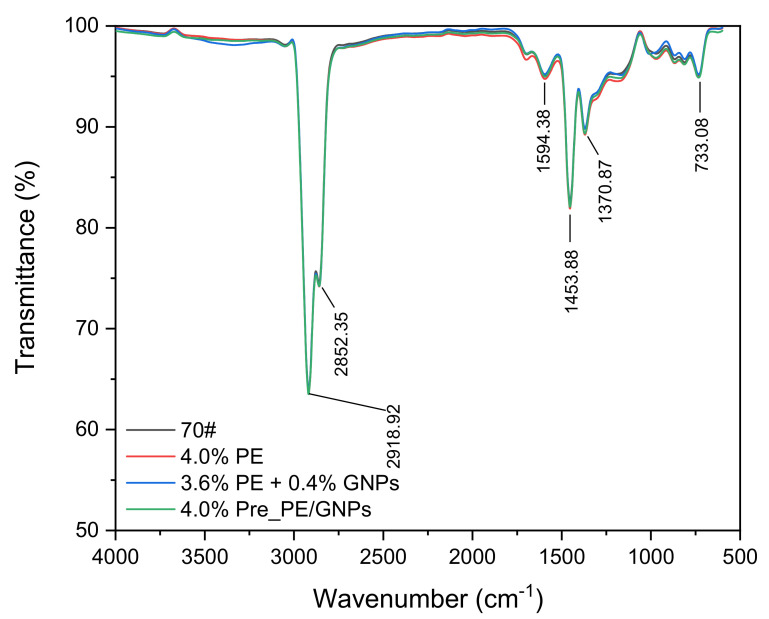
FT-IR spectra of asphalt binder samples.

The rheological properties of PE/GNPs composite modified asphalt discussed above depend largely on the microstructure and chemical properties of the material system. In order to further understand the different properties of various PE/GNPs composite modified asphalts and their interaction mechanisms, the chemical composition and functional group distribution of PE/GNPs modified asphalts were examined by means of Fourier transform infrared (FT-IR) spectroscopy. As shown in Figure 9, the characteristic absorption peaks of the asphalt binder samples were mainly concentrated at 2918.92 cm^−1^, 2852.35 cm^−1^, 1594.38 cm^−1^, 1453.88 cm^−1^, 1370.87 cm^−1^, and 733.08 cm^−1^, among which the peaks at 2918.92 cm^−1^, 2852.35 cm^−1^ 1 are caused by the stretching vibrations of aliphatic C-H bonds; the absorption bands at 1594.38 cm^−1^, 1453.88 cm^−1^, 1370.87 cm^−1^ and 733.08 cm^−1^ correspond to the vibrations of benzene rings and their substituents [37], and 814.2 cm^−1^ corresponds to the fingerprint absorption peak of PE [15]. Among the three asphalt binders mentioned above, the intensity of the absorption peaks of PE modified asphalt was relatively larger due to the more sensitive response of PE as an organic modifier in the FT-IR spectrum, while GNPs, as a typical inorganic filler, introduced into the asphalt matrix would significantly reduce the intensity of the absorption peaks caused by the C–H bond. Combining the test data of the three asphalts, it can be seen that no new absorption peaks appeared in the PE modified asphalt after the addition of the appropriate amount of GNPs, and only the intensity of individual characteristic peaks differed, which indicated that GNPs in PE modified asphalt mainly produced physical modification, thus enhancing the rheological properties of the composite modified asphalt.

## 4. Conclusions

1. PE and GNPs synergistically improved the high-temperature properties of asphalt binders (increased softening point and viscosity); the premixed PE/GNPs masterbatch modified asphalt exhibited better compatibility compared with 3.6% PE + 0.4% GNPs;
2. The PE/GNPs composite additive effectively enhanced the rheological properties of the asphalt binder, in which 3.6% PE + 0.4% GNPs modified asphalt could significantly improve the high-temperature rutting resistance of the asphalt binder. Comparison of the composite modified asphalt binder prepared by the two processes revealed that the 4.0% Pre_PE/GNPs composite modified asphalt presented better medium-temperature fatigue performance and low-temperature cracking resistance;
3. Field emission scanning electron microscopy revealed homogeneous dispersion of PE/GNPs composite modifier in the asphalt matrix, where the premixed PE/GNPs masterbatch exhibited a more compact structure and better compatibility with asphalt binder;
4. Fourier transform infrared spectroscopy confirmed that no new functional groups or chemical bonds appeared in the PE/GNPs composite modified asphalt, and the rheological properties of base asphalt incorporated with GNPs and PE were enhanced by physical modification.

Given that the polyethylene/graphene composite modifier indeed improved the rheological properties of asphalt binder, the subsequent research should focus on the effects of aging (short- and long-term aging) on the performance of PE/GNPs modified asphalt and its mechanism of action. In addition, the transfer and evolution of functional properties of GNPs (excellent electrical, thermal conductivity, and barrier properties) in asphalt and mixtures need to be further explored for the development of new asphalt pavements. The results of this study can lay a solid theoretical foundation for the application of graphene/polyethylene composite modification technology in road engineering and guide the application practice of composite modifiers in asphalt binders.

## Figures and Tables

**Table 1:** The physical properties of base asphalt.

Items	Results	Requirements
Penetration (25 °C) (0.1 mm)	66.80	60–80
Softening point (°C)	47.60	≥45
Ductility (15 °C) (cm)	112	≥100
Density (15 °C) (g/cm^3^)	1.035	measurement
Dynamic viscosity (60 °C) (Pa·s)	data	≥180

**Table 2:** The physical properties of GNPs.

Properties of GNPs		Properties of PE	
Items	Test Results	Items	Test Results
Average thickness (nm)	<3	Density (g/cm^3^)	0.914
Slice layer size (μm)	5–10	Crystallinity (%)	75
Specific surface area (m^2^/g)	40–60	Melting point (°C)	126
Ash content (%)	<3	Melt flow index (MFI) (g/10 min)	1.8
Film conductivity (S/cm)	>700	Break elongation (%)	525
Tap density (g/cm^3^)	0.02–0.03	Break strength (MPa)	18.4

**Table 3:** Summary of test parameters for multiple stress creep recovery of asphalt binder.

Types of Asphalt	*R*_0.1_/%	*R*_3.2_/%	*J_nr_*_0.1_/kPa^−1^	*J_nr_*_3.2_/kPa^−1^	*J_nr-diff_*/%
70#	3.14	0.15	2.88	4.55	69.93
4.0% PE	8.24	4.62	0.72	1.14	57.68
3.6% PE + 0.4% GNPs	27.20	8.17	0.63	0.99	55.85
4.0% Pre_PE/GNPs	12.21	4.75	1.15	1.74	51.61

## Data Availability

The data presented in this study are available on request from the corresponding author.
